# Comprehensive spectral identification of key intermediates to the final product of the chiral pool synthesis of radezolid

**DOI:** 10.1186/s13065-017-0309-x

**Published:** 2017-08-09

**Authors:** Katarzyna Michalska, Elżbieta Bednarek, Ewa Gruba, Kornelia Lewandowska, Mikołaj Mizera, Judyta Cielecka-Piontek

**Affiliations:** 1 Department of Antibiotics and Microbiology, National Medicines Institute, Chelmska 30/34, 00-725 Warsaw, Poland; 2 Department of Counterfeit Medicinal Products and Drugs, National Medicines Institute, Chelmska 30/34, 00-725 Warsaw, Poland; 3 Department of Molecular Crystals, Institute of Molecular Physics of the Polish Academy of Sciences, Smoluchowskiego 17, 60-179 Poznan, Poland; 4 Department of Pharmaceutical Chemistry, Poznan University of Medical Sciences, Grunwaldzka 6, 60-780 Poznan, Poland

## Abstract

Radezolid (RAD, **12**), biaryl oxazolidinone, was synthesised with small modifications according to the methods described in the literature. The pharmacological activity is observed only for (*S*)-enantiomer, therefore its synthesis is oriented towards obtaining a single isomer of required purity and desired optical configuration. The intermediate products of RAD synthesis were characterised using ^1^H- and ^13^C-NMR, as well as the 2D correlation HSQC and HMBC (**2**, **5**, **9**, **10**), furthermore studied using infrared radiation (FT-IR), Raman scattering (**3**, **5**, **9**), and electronic circular dichroism (ECD) (**5**, **12**) spectroscopy. Each technique provides a unique and specific set of information. Hence, the full spectral characteristics of key intermediates obtained from the chiral pool synthesis to the finished product of RAD were summarised and compared. For a more accurate analysis, and due to the lack of reliable and reproducible reference standards for intermediate products, their vibrational analysis was supported by quantum chemical calculations based on the density functional theory (DFT) utilising the B3LYP hybrid functional and the 6-311G(d,p) basis set. Good agreement was observed between the empirical and theoretical spectra.

**Electronic supplementary material:**

The online version of this article (doi:10.1186/s13065-017-0309-x) contains supplementary material, which is available to authorized users.

**Graphical abstract:**
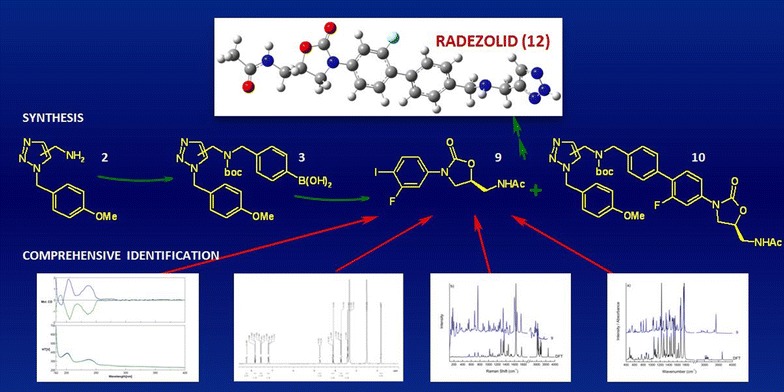
Comprehensive spectral identification (ECD, NMR, FT-IR, Raman) of key intermediates of the chiral pool synthesis of radezolid.

## Background

Radezolid (RAD) N-{[(5*S*)-3-[3-fluoro-4-(4-{[(1H-1,2,3-triazol-5-ylmethyl)amino]methyl}phenyl)phenyl]-2-oxo-1,3-oxazolidin-5-yl]methyl}acetamide belongs to second generation oxazolidinones, after its predecessors, such as linezolid and tedizolid. Oxazolidinones are, undoubtedly, the most promising, prospective, and anticipated class of antimicrobial agents, taking one of the burning issues in human health; namely, the rapid spread of multidrug-resistant pathogenic bacteria. Thus, the activity of RAD against linezolid-resistant staphylococci as well as against causative agents of community-acquired pneumonia, such as *Haemophilus influenzae* and *Moraxella catarrhalis* [1] seems to be crucial for the widely understood clinical interest. RAD has completed two phase 2 clinical trials (http://clinicaltrials.gov), the first on community-acquired pneumonia, and the second on uncomplicated skin and skin-structure infections; however, it still remains in the clinical development stage [2].

**Fig. 1:**
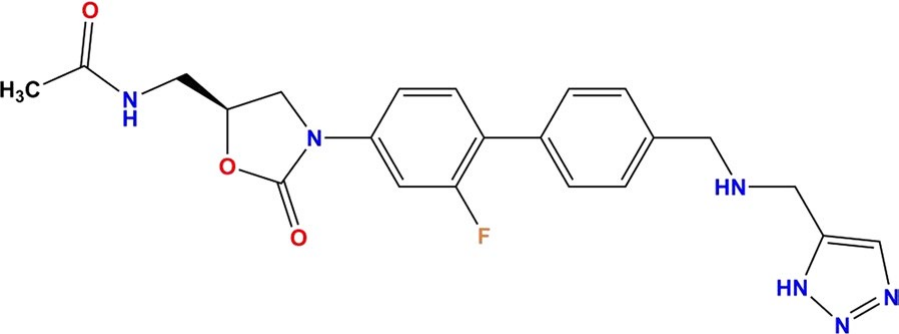
Molecular structure of radezolid

From a chemical point of view, the molecular structure of RAD can be divided into the following building units, as seen in Fig. 1: triazole ring, methylaminomethyl link, biaryl ring system, oxazolidinone ring, and acetamide fragment. RAD is completely synthetic, and possesses a single stereocentre at position C5 of the oxazolidinone ring.

When considering the safety of pharmacotherapy, the development of both efficient optically pure synthesis and methods for their control is essential to ensure the quality, safety and efficacy of chiral drugs, especially due to the fact that only one of the enantiomers generally possesses the desired therapeutic activity and favourable pharmacological profile, while the second is inactive and may contribute to greater toxicity.

**Scheme 1:**
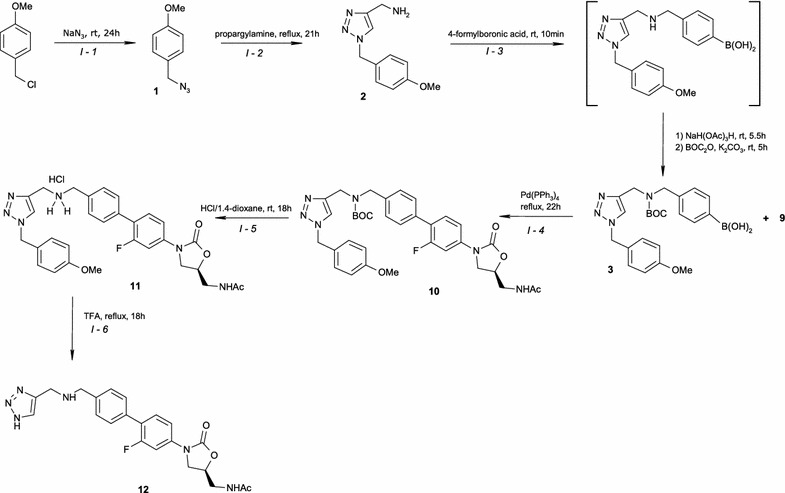
Pathways of RAD synthesis

**Scheme 2:**
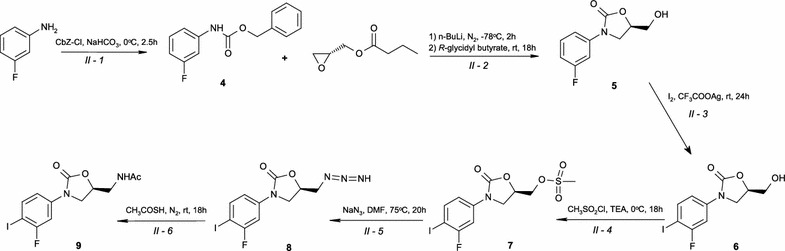
Pathways of intermediate **9** synthesis

The pharmacologically active isomer of RAD was synthesised based on the literature with several changes in order to enhance the overall efficiency of its synthesis (Schemes 1, 2) [3–8]. However, RAD may be synthesised by different pathways of preparation; thus, full characterisation of key intermediates as well as RAD are crucial for the quality control, considering the safety of pharmacotherapy, as stated above. Therefore, the aim of this work was, comprehensive spectral characterisation and the identification of important intermediate compared to the finished product of RAD [9] by spectroscopic methods (FT-IR, Raman, ECD, ^1^H- and ^13^C-NMR).

With reference to the implementation of the purpose, various spectroscopic methods have been used, which provide distinct, usually fragmentary, information regarding the absorbing molecules. However, those information complement each other and confirm itself, giving the most complete overall view of the molecule. In the IR spectra, strong absorption bands are derived from vibrations of polar groups such as, O–H, N–H, and C=O. In contrast, in the Raman spectra, there are active vibrations, where changes in polarisability bond during normal vibrations are observed, with no change in the electrical dipole moment, as for the infrared bands. Thus, the Raman active vibrations are intense for nonpolar moieties, such as homonuclear fragment like C=C, N–N, and S–S, while the vibrations of a highly polar moiety are usually weak. Hence, Raman scattering complements IR spectroscopy. It is worth pointing out that those techniques are valuable analytical tools for the analysis of “specific” drugs, e.g. labile drugs or those with required chiral purity [10–14]. Similar approaches to the identification of oxazolidinone analogues have been reported in the literature for linezolid and tedizolid [15–17]. However, so far, no reports have described the comprehensive characterisation of intermediate products which are important for the chiral pool synthesis of RAD by spectroscopic methods.

In addition, results from vibrational spectroscopy have been complemented by the analysis of chemical shifts in the NMR spectra. The application of NMR methods for control of the synthesis of pharmaceutical substances belongs to a complete approach, due to these tests providing knowledge about chemical bonds together with compositional information for macromolecular products of synthetic origin [18]. NMR combined with vibrational spectroscopy gives a full insight into the structure of the investigated intermediates compared with the finished product.

However, NMR and vibrational spectroscopy have not allowed the enantiopurity to be determined directly, or enantiomers to be distinguished. ECD, as chiroptical method, is one of the most sensitive spectral techniques commonly used for the study of control chiral purity [19].

## Results and discussion

The results’ presented in this paper were discussed with particular focus on addressing following research problem: evaluation of the identification of key intermediate products in relation to characterisation of the finished product [9], including estimation of its chiral purity.

The most important step in the synthesis of RAD (described in detail in Additional file 1) is the cross-coupling reaction of the boroorganic acid derivative (**3**) and iodooxalidinone derivative (**9**) catalysed by tetrakis(triphenylphosphine)palladium(0) based on Suzuki reaction mechanism, preceded by preparing those two building block compounds, which leads to the molecule described as (**10)**.

The protected ({[[1,2,3] triazol-4-ylmethyl]amino}methyl)phenylboronic acid moiety (**3**) was obtained from 4-methoxybenzyl chloride on which the triazole ring was built.

*N*-{[(5*S*)-3-(3-fluoro-4-iodophenyl)-2-oxo-1,3-oxazoilidin-5-yl]methyl}acetamide (**9**) was prepared from *R*-glycidyl butyrate >98% as a chiral carrier, and a well-known starting material to use with the reaction of a carbamate, *N*-carboxyloxy-3-fluoroaniline, an oxazolidinone ring, which allowed only one, enantiomerically pure, desired oxazolidinone derivative to be obtained, in four simple steps.

**Fig. 2:**
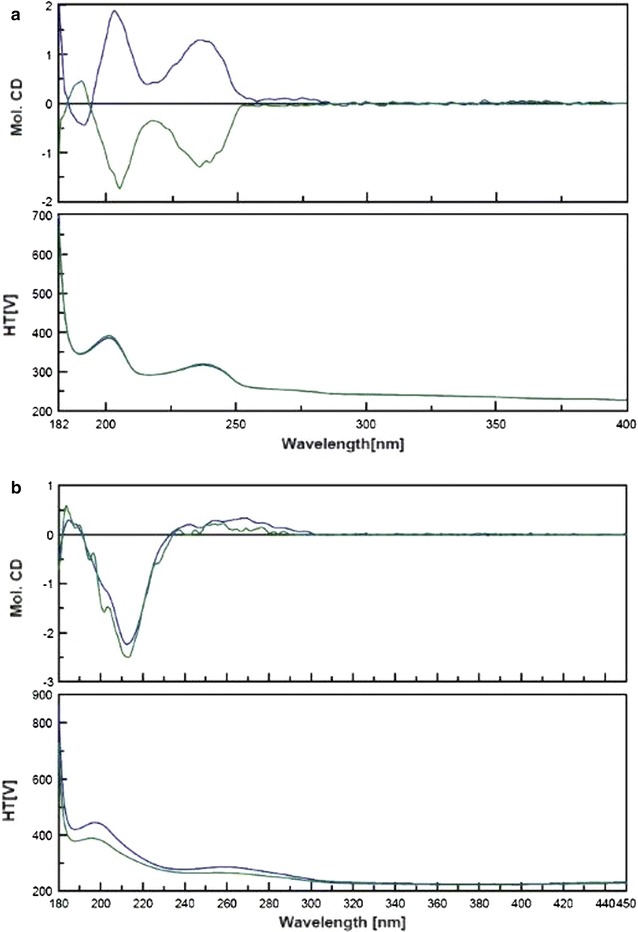
The comparison of electronic circular dichroism spectra of **a** (*S*)- (*blue*) and (*R*)-**5** (*green*), and **b** synthesised (*S*)-radezolid (*blue*) and reference material (*green*)

Major changes to the synthesis of RAD proposed by Gravestock and co-workers [5] focused on the step leading to compound **10**. The 0.01 eq amount of palladium catalyst was not sufficient to initiate a coupling reaction; increasing the amount to 0.1 eq allowed **10** to be obtained with good yield. Other changes included the higher amounts of solvents and the addition of a new one, the extension of reaction time and temperature and the new procedure involving crystallisation of the final product **12** [9]. Chiral pool synthesis allowed the finished product to be obtained at a suitable chiral purity. The ECD spectroscopy was used as a reference measurement method; undoubtedly, this is the most appropriate spectral technique for describing chiral phenomena. Firstly, a comparison of the ECD spectra of the synthesised (*S*)- and (*R*)-enantiomers of **5** were performed to confirm a lack of inversion of the chiral centre, at this crucial, oxazolidinone ring closure stage. These mirror image isomers of compound **5** are presented in Fig. 2a. For the (*R*)-isomer of **5**, which leads to (*S*)-RAD (**12**), a positive Cotton effect at 191.6 nm was observed, while a negative Cotton effect was noticed at 203.4, 236.8, and 275.4 nm. Secondly, comparison of the ECD spectra of the finished product (**12**) to the reference material of RAD demonstrated that the differences found in the shapes of the spectra curves were not significant, which confirmed the chiral purity of the synthesised product as a consequence, as seen in Fig. 2b [9]. RAD showed a positive Cotton band with its maximum at 185.0 versus 183.6 nm, and 268.0 versus 258.3 nm, as well as a negative band with the maximum at 212.4 versus 213.3 nm for the synthesised product and reference material, respectively. Those observations suggested that the synthesised RAD did not contain any impurities of the chromophore structure. If the sample was contaminated by impurities, it would lead to a change in the position of the absorption maxima and the shape of the spectra, which was not observed in this particular case. Only a shift at 260 nm of about 10 nm to the spectra of reference material may indicate that this shift was obtained for lower and wider Cotton bands in this spectrum region; therefore, the maximum value is blurrier, hence the difference. Comparative analysis showed a high level of compliance with spectra reference material.

What is important, Okuom and co-workers [20] have presented qualitative evaluation of enantiopurity by ECD in the absence of chiral selectors normally required in different separation techniques. The ECD spectra of both enantiomers were determined and, than plotting the differential extinction coefficient (Δε) versus enantiopurity at the wavelength of maximum amplitude were performed. However appropriate quantity of (*S*)- and (*R*)- enantiomers of reference materials are needed. In our experiments we compared synthetized (*S*)-radezolid to the reference material of (*S*)-isomer, and based on the compatibility of the spectrum we have requested chirality. The absence of (*R*)-radezolid as a reference substance made it impossible to carry out the experiment proposed by Okuoma et al.

On the other hand, in case of high purity (99 +% to 95% ee) of studied compound, virtually identical ECD spectra could be obtained, so to determine the chiral purity of radezolid, capillary electrokinetic chromatography modified by cyclodextrin, realized by Michalska and co-workers may be proposed [21]. However, it should be stressed out, that only (*S*)-enantiomer is active pharmaceutically, therefore in the quality control majority of studies will be focused on its identification.

Simultaneously, in our spectroscopic analysis of the identification of intermediate and final products, vibrational spectroscopy, and complementary tools such as NMR experiments have been employed. By using FT-IR and Raman spectroscopy, 4-({t-butoxycarbonyl-[1-(4-methoxybenzyl)-1H- [1–3] triazol-4-ylmethyl]amino}methyl)phenylboronic acid, (**3**), (5*R*)-3-(3-fluorophenyl)-5-hydroxymethyl-2-oxooxazolidine (**5**), and *N*-{[(5*S*)-3-(3-fluoro-4-iodophenyl)-2-oxo-1,3-oxazoilidin-5-yl]methyl}acetamide (**9**) were identified. For a more accurate analysis and due to the lack of reliable and reproducible references, the identification of intermediate products **5** and **9** was supported by DFT using a B3LYP hybrid functional with a Quadruple Zeta Valence plus Polarisation function (QZVP) basis set. The calculated and experimental FT-IR, and Raman spectra for **5**, **9**, and **3** have been presented in Additional file 1. The comparison of the frequencies calculated by DFT–B3LYP method with the experimental values reveals an overestimation of the calculated vibrational modes due to neglect of anharmonicity in the real system. Normally, the overestimation of unscaled frequencies in comparison to observed frequencies was prominent only in the higher frequency region. Better approximation of the observed fundamental frequencies was achieved for the B3LYP/6-311G(d,p) calculations than the B3LYP/6-31G(d,p) results. Therefore, it is customary to scale down the calculated harmonic wavenumber in order to improve the conformity with the experimental values. The harmonic vibrational frequencies were scaled by 0.967 for B3LYP/6-311G(d,p). Three key intermediate products of the chiral pool synthesis (**3**, **5**, **9**) discussed in this paper possess many common bands corresponding to the same vibrations (Table 1). Very often, they are shifted, even 20 cm^−1^; thus, e.g. the bands in IR absorption spectra, which appeared as strong bands at 750/753/760 cm^−1^, are related to the characteristic bending vibration out of plane of the C–O–N bonds in the oxazolidinone ring for RAD, **5**, and **9**, respectively. For RAD, this band has an additional component associated with the bending vibration out of plane of C–C–N bonds in 1,2,3-triazole ring and the C–N–C [(methyl)amino]methyl group. The bands related to the stretching vibration of the C–O, and C–C, as well C–N bonds in oxazolidinone ring and stretching vibration of the C–F bond in F-phenyl ring for those three structures are also visible at 872, 906, 1036, 1081, 1202, 1253 cm^−1^ and 860, 882, 1012, 1083, 1196, 1298 cm^−1^ and 869, 899, 1029, 1093, 1201, 1274 cm^−1^ for RAD, **5**, and **9**, respectively. The band located at 1329/1340/1338 cm^−1^ in RAD, **5**, and **9**, respectively, is related to the stretching vibration of the C–N bond between oxazolidinone and F-phenyl rings. For RAD, this band has an additional component corresponding to the stretching vibration of the C–N bond in the 1,2,3-triazole ring too, as band at 1417 cm^−1^ for RAD. One of the strongest bands, both in IR absorption and Raman scattering spectra, was related to the stretching vibration of the C=C and C=O bonds in phenyl, F-phenyl, and oxazolidinone rings, and they were located at 1577, 1629, 1754 and 1590, 1612, 1725 cm^−1^, as well at 1570, 1596, 1749 cm^−1^ for RAD, **5** and **9**, respectively. The stretching vibration of the C–H bonds are also visible for all three samples, and are located for example at 2960/2953/2957 cm^−1^ for RAD, **5**, and **9**, respectively. The bands related to the vibration in the methylacetamide group are also visible for two compounds, RAD and **9**. For example, the band located at about 1530 cm^−1^ is related to the stretching vibration of the N–H bond, and the band at about 1676 cm^−1^ is associated with the characteristic stretching vibration of the C=O bond, whereas the band at about 3419/3410 cm^−1^ for RAD, and **9**, respectively, is related to the stretching vibration of the N–H bond as well. For **5**, sample characteristic bands primarily related to the stretching vibration of the C–O bond, bending and stretching vibration of C–H and O–H bond in the COH_3_ group were also noticed. They are located at 1040, 1233, 2871 and 3521 cm^−1^. RAD was characterised by the bands at 798, 975 and 1442 cm^−1^. The first band is associated with the out of plane bending vibration of the N–H bonds in 1,2,3-triazole ring, the second corresponds to the C–N–C [(methyl)amino]methyl group and the third band responds to the in-plane bending vibration of the same bonds. Otherwise, the band at 975 cm^−1^ is related to the bending vibration of the C–N–C bond in the [(methyl)amino]methyl group.

**Table 1:** Selected characteristic vibrionic features of **RAD**, **9** and **5** in theory with application of 6-311G(d,p) basis and experiment bands of **9** and **5**

ν_exp.IR_			$v_{{\varvec{exp}.\varvec{R}}}$			Theory DTF			Bands assignment
RAD^a^	9	5	RAD^a^	9	5	RAD^a^	9	5	
	542	522		547	522		552	528	Def. F-phenyl ring
601	594	594		592			602	609	Def. all molecule
		666		676	667		680	679	Def. oxazolidinone ring
		681		693			707	699	C–H *b op* in F-phenyl ring
	745	737	743	750	737	744	757	751	C–C–O *b* in oxazolidinone ring + C–C–N *b* in triazole ring and in methyloacetamide group
750	760	753			752	777	763	763	N–H *w* in triazole ring
		783						785	C–H *b* op in F-phenyl ring
798						803			N–H *op* in 1,2,3-triazole ring + N–H *op* in link + C–H *w* in F-phenyl ring
838	848	837				851	852	857	C–H *w* in 1,2,3-triazole ring
872	869	860		870		890	881	866	Breathing oxazolidinone and F-phenyl rings
		874						857	C–H *b op* in F-phenyl ring
906	899	882		902		916	926	908	Def. F-phenyl ring + C–O *s* in oxazolidinone ring
	957	930					987	964	C–H *r* in CH_2_ group
975			976			969			C–N–C *b* in link + C–H *t* in link + C–C *s* in 1,2,3-triazole ring
1020		996	1020	1002		1014	1023	1013	C–C *s* in oxazolidinone ring
1036	1029	1012		1028	1012	1048	1053	1034	C–O *s* in oxazolidinone ring + C–F *s* + C–H *b* in F-phenyl ring + C–J *s* + C–C *s* in oxazolidinone ring
		1040						1043	C-O *s* in COH_3_
	1042						1073		C–C *s* between in oxazolidinone ring and methylacetamide group
			1109			1068			C–N–C *b* in link + C–H *t* in methylacetamide group
	1117	1097	1121			1083	1112	1098	C–H *r* in F-phenyl ring + C–N s in oxazolidinone ring
	1133	1121			1118			1122	C–H *b*
1136						1130			C–H *sc* in F-phenyl ring + C–F *s* in F-phenyl ring
	1164	1147		1154	1148		1150	1142	C–O *s* in oxazolidinone ring + C–H *b*
		1168						1186	C–H *b* in F-phenyl ring + C–F *s* + C–N *s*
1202	1201	1196		1197		1186	1208	1204	C–N *s* in oxazolidinone ring + C–H *r* + N–H *r* in 1,2,3-triazole ring
		1233			1230			1228	C–N *s* + C–H *sc* in oxazolidinone ring + O–H *b*
1225	1227					1216	1230		N–N *r* in methylacetamid group + C–C *s* in methylacetamid group + C–H *r* F-phenyl ring + C–H *w* in methylacetamid group
1230						1232			C–C *s* between phenyl ring and link + C–N *s* in oxazolidinone ring + C–F *s* in F-phenyl ring + C–H *t* in oxazolidinone ring
	1249	1280		1246	1280		1285	1307	C- F-phenyl ring + C–H *b* + C–N *s* between oxazolidinone and F-phenyl rings + C–N *s* and N–H *b* in methylacetamide group
1253	1274	1298			1299	1251	1306	1322	C–H *t* in methylacetamid group + C–H *sc* in oxazolidinone ring + C–H *r* in phenyl and F-phenyl ring
1293						1273			C–C *s* in phenyl and F-phenyl ring + C–H *w* in phenyl ring and methyloacetamide group
				1304			1277		C–C *s* between phenyl and F-phenyl ring + C–C *s* in phenyl and F-phenyl ring
1329	1338	1340	1326	1335	1341	1307	1358	1369	C–N *s* between 1,2,3-triazole and oxazolidinone ring + C–N *s* in 1,2,3-triazole ring + C–H *r* in phenyl ring
	1357	1380		1367	1380		1385	1384	C–H *b* in oxazolidinone ring
1417	1413	1419	1416	1415	1421	1433	1427	1434	N–H *r* in 1,2,3-triazole ring + methyloacetamide group
1442						1443			N–H *r* in methyloacetymide group
	1479	1495		1480	1497		1513	1518	C–H *sc* in oxazolidinone ring + C–H *r* in F-phenyl ring + C–N *s* between oxazolidinone and F-phenyl rings
1530	1528		1530			1502	1547		C–C *s* between phenyl and F-phenyl ring + C–H *r* in phenyl and F-phenyl ring
1577	1570	1590			1593	1557	1597	1616	C=C *s* + C–C *s* in phenyl and F-phenyl rings
1629	1596	1612	1617	1596	1612	1598	1631	1645	C=C *s* in phenyl and F-phenyl rings
1676	1672			1659		1703	1681		C=O *s* in methylacetamide group
1754	1749	1725		1748	1725	1786	1762	1756	C=O *s* in oxazolidinone ring
		2871						2998	C–H *s* in COH_3_
2928		2928		2894		3023	3028	3029	C–H *s* in oxazolidinone ring
2960	2957	2953					3075	3069	C–H *s* in oxazolidinone ring
		2983		2980			3106	3106	C–H *s* in oxazolidinone ring
3114	3099	3110				3248	3249	3248	C–H *s* in F-phenyl ring
3419	3410			3408		3637	3635		N–H *s*
		3521						3826	O–H *s*

*s* stretching, *b* bending, *w* wagging, *t* twisting, *r* rocking, *sc* scissoring, *op* outside of the plane, *ip* in plane, *asym* asymetric, *sym* symetric

^a^Data included in the manuscript concerning application of spectroscopic methods (FT-IR, Raman, ECD and NMR) in studies of identification and optical purity, Spectrochimica Acta Part A: Molecular and Biomolecular Spectroscopy [9]

Due to the lack of a theoretical spectrum, analysis of the key intermediate product **3** was based on knowledge and experience of the positions of characteristic bands [22, 23]. The basic structure of **3** consists of two rings: the 1,2,3-triazole ring and the phenyl ring linked by the [(methyl)amino]methyl group, which is common with the RAD structure. In the absorption of IR and Raman scattering spectra, many bands that are related to vibration of the same bands as in RAD were observed. For example, the most characteristic and strong band was visible in Raman spectrum at 1616 cm^−1^, associated with the stretching vibration of the C=C bond in the phenyl ring. The same band in the absorption of IR spectrum was observed at 1611 cm^−1^. The band related to the stretching vibration of the C–C bonds in the phenyl ring was also located at 1304 cm^−1^ in the Raman spectrum, whereas in the FT-IR spectrum, the band related to the stretching vibration of the C–C bond in the 1,2,3-triazole ring was located at 983 cm^−1^. The stretching vibration of the C–N bonds in 1,2,3-triazole ring and [(methyl)amino]methyl group were shifted by a few cm^−1^ and located at 1408 and 1250 cm^−1^, respectively, whereas the band corresponding to the rocking vibration of the N–H located to the RAD at 1442 cm^−1^ was shifted to 1453 cm^−1^ in the FT-IR spectrum and to 1461 cm^−1^ in the Raman spectrum. That strong shift due to the lack of vibration of N–H bonds in the [(methyl)amino]methyl group was noticed, which did not exist in **3**. The small band related to the bending vibration of the C–C–N bonds in the 1,2,3-triazole ring in the spectra of **3** was also visible, and located at about 750 cm^−1^. On the other hand, the bands associated with the characteristic vibration of **3** were also observed. For example, the band at 1690 cm^−1^ was related to the stretching vibration of the C=O bond, and was present only in the spectrum for **3**. Many bands related to the rocking, scissoring and bending vibrations of the C–H bonds were observed in the range 1500–800 cm^−1^ in FT-IR, and Raman spectra, whereas the bands corresponding to the stretching vibration of these bonds were noticed above 3000 cm^−1^. However, any discrepancies observed in the spectra have been related to imperfections of computational modelling of the isolated molecule. Implications of the computational capability of the QZVP basis set has prevented theoretical calculations of the spectra of intermediate product **3** due to the presence of boron atoms. Hence, the significance of the complexity of block **3** has made computations too time-consuming.

In the parallel stage, ^1^H NMR spectra for samples of intermediates were obtained straight from the synthesis steps. The spectra of reaction mixtures, crude or partially purified (e.g. evaporation of the solvents, the removal of the substrates), were recorded. The section on purification of samples after subsequent steps of synthesis was described in detail in Additional file 1. If the analysis of ^1^H NMR spectra of these samples indicated the presence of the expected product, then ^13^C NMR and 2D correlation HSQC and HMBC spectra were additionally performed. Analysis has been mostly limited to identifying the characteristic changes in the NMR spectra, which could confirm the desirable direction of the reaction, i.e. chemical shifts of NMR signals of functional groups of the reactants which are responsible for the reaction course were observed. If the reduction in intensity or disappearance of the above-mentioned signals was observed with the simultaneous appearance of new signals specific to a new molecule, it was possible to confirm the proper direction of the reaction (**3**).

Full analysis of the NMR spectra were carried out for purified samples (**2**, **5**, **9**, **10**). The signals in the ^1^H and ^13^C NMR spectra of studied compounds were assigned to the protons and carbon atoms in the appropriate structural fragments using general knowledge of the chemical shift dispersion. The assignment of signals to proton and carbon atoms of CH, CH_2_ or CH_3_ groups was confirmed by the ^1^H{^13^C} HSQC experiments. The ^1^H{^13^C} gHMBC spectra were used as a final and unambiguous tool assign NMR signals, including the quaternary carbon atom resonances.

The ^1^H and ^13^C NMR data (chemical shifts, δ [ppm], multiplicity, coupling constants: proton-proton *J*_*HH*_, proton–fluorine *J*_*HF*_, carbon–fluorine *J*_*CF*_ [Hz] and HMBC correlations) are given in Tables 2, 3, 4 and 5 for compounds **2 (Step I-2)**, **5** (**Step II-2**), **9 (Step II-6), 10 (Step I-4)**, as depicted in Schemes 1 and 2.

**Table 2:** 1D and 2D-NMR data of **2** in DMSO (2.50 ppm-^1^H/39.4 ppm-^13^C) at 500 MHz


Atom position	δ_H_ [ppm], multiplicity, *J*_HH_ [Hz]^a^	δ_C_ [ppm]	**HMBC** correlations **(H→C)** ^a,b^
**4**	–	149.7	–
**5**	7.86 (bs, 1H, triazole–C**H**)	121.5	**4, 7′** (w)
**6**	3.73 (bs, 2H, C**H**_**2**_–NH_2_)	37.0	**5, 4**
**7**	5.46 (s, 2H, Ar–C**H**_**2**_)	52.1	**2′/6′, 1′, 5**
**1′**	–	128.0	–
**2′/6′**	7.29 (d, 2H, *J* = 8.7 Hz)	129.5	**4′, 2′/6′,** 7′**, 3′/5′**
**3′/5′**	6.92 (d, 2H, *J* = 8.7 Hz)	114.0	**1′, 3′/5′, 2′/6′, 4′**
**4′**	–	159.0	–
Ar–O–**CH**_**3**_	3.74 (s, 3H, Ar–OC**H**_**3**_)	55.0	**4′**

^a^ *s* singlet, *bs* broad singlet, *d* doublet, *w* weak

^b^This column gives the carbon atoms showing correlation with a given proton

**Table 3:** 1D and 2D-NMR data of **5** in DMSO (2.504 ppm-^1^H/39.4 ppm-^13^C) at 500 MHz


Atom position	δ_H_ [ppm], multiplicity, *J*_HH_or *J*_HF_ [Hz]^a^	δ_C_ [ppm], *J*_CF_ [Hz]^a^	**HMBC** correlations **(H→C)** ^a,b^
**2**	–	154.3	–
**4**	3.84 (dd, 1H, *J* = 8.9, 6.2 Hz) 4.09 (dd, 1H, *J* = 8.9, 9.1 Hz)	45.9	**2**, **5**, **6** **2**, **5**, **6**
**5**	4.72 (m, 1H);	73.2	–
**6**	3.56 (ddd, 1H, *J* = 12.4, 5.8, 4.0 Hz) 3.68 (ddd, 1H, *J* = 12.4, 5.4, 3.4 Hz)	61.5	**4** **4**, **5** (w)
**OH**	5.23 (dd, 1H, *J* = 5.4, 5.7 Hz)	–	**5**, **6**
**1′**	–	140.1 (d, *J*_*CF*_ = 11.0 Hz)	–
**2′**	7.53 (ddd, 1H, *J* = 11.95, 2.5, 2.2 Hz)	104.6 (d, *J*_*CF*_ = 27.1 Hz)	**3′**, **6′**, **4′**, **1′**
**3′**	–	162.2 (d, *J*_*CF*_ = 241.5 Hz)	–
**4′**	6.95 (dddd, 1H, *J* = 8.4, 8.4, 2.6, 0.9 Hz)	109.6 (d, *J*_*CF*_ = 21.2 Hz)	**3′**, **6′**, **2′**
**5′**	7.43 (ddd, 1H, *J* = 8.3, 8.3, 6.8 Hz)	130.5 (d, *J*_*CF*_ = 9.6 Hz)	**3′**, **1′**, **6′**(w), **2′**(w)
**6′**	7.34 (ddd, 1H, *J* = 8.3, 2.2, 0.9 Hz)	113.3 (d, *J*_*CF*_ = 2.8 Hz)	**2′**, **4′**

^a^ *d* doublet, *dd* doublet of doublets, *ddd* doublet of doublets of doublets, *dddd* doublet of doublets of doublets of doublets, *m* multiplet, *w* weak

^b^This column gives the carbon atoms showing correlation with a given proton

**Table 4:** 1D and 2D-NMR data of **9** in DMSO (2.504 ppm-^1^H/39.4 ppm-^13^C) at 500 MHz


Atom position	δ_H_ [ppm], multiplicity, *J*_HH_ or *J*_HF_ [Hz]^a^	δ_C_ [ppm], *J*_CF_ [Hz]^a^	**HMBC** correlations **(H→C)** ^a,b^
**2**	–	153.8	–
**4**	3.73 (dd, 1H, *J* = 9.3, 6.6 Hz) 4.11 (dd, 1H, *J* = 9.0, 9.0 Hz)	47.0	**2**, **5**, **6** **2**, **5**, **6**
**5**	4.72 (m, 1H)	71.7	**2**
**6**	3.41 (dd, 1H, *J* = 5.7, 5.7 Hz)	41.2	**8**, **5**, **4**
N**H**COCH_3_	8.23 (t, 1H, *J* = 5.7)	–	**8**, **6**
**8**	–	169.9	–
**9**	1.83 (s, 3H)	22.3	**8**
**1′**	–	140.3 (d, *J*_*CF*_ = 10.5 Hz)	–
**2′**	7.55 (dd, 1H, *J* = 10.9, 2.3 Hz)	105.2 (d, *J*_*CF*_ = 29.8 Hz)	**3′**, **6′**, **4′**, **1′**
**3′**	–	161.1 (d, *J*_*CF*_ = 240.6 Hz)	–
**4′**	–	74.0 (d, *J*_*CF*_ = 21.2 Hz)	–
**5′**	7.83 (dd, 1H, *J* = 8.7, 7.7 Hz)	139.0 (d, *J*_*CF*_ = 3.4 Hz)	**3′**, **1′**, **4′**, **2′**(w)
**6′**	7.19 (dd, 1H, *J* = 8.8, 2.4 Hz)	115.5 (d, *J*_*CF*_ = 2.9 Hz)	**2′**, **4′**

^a^ *s* singlet, *d* doublet, *dd* doublet of doublets, *t* triplet, *m* multiplet, *w* weak

^b^This column gives the carbon atoms showing correlation with a given proton

**Table 5:** 1D and 2D-NMR data of **10** in DMSO (2.50 ppm-^1^H/39.4 ppm-^13^C) at 500 MHz


Atom position	δ_H_ [ppm], multiplicity, *J*_HH_ or *J*_HF_ [Hz]^a^	δ_C_ [ppm], *J*_CF_ [Hz]^a^	**HMBC** correlations **(H→C)** ^a,c^
**2a**	–	153.9	–
**4a**	3.79 (dd, 1H, *J* = 9.1, 6.4 Hz) 4.17 (dd, 1H, *J* = 9.1, 9.1 Hz)	47.1	**2a**, **5a**, **6a** **2a**, **5a**, **6a**
**5a**	4.77 (m, 1H)	71.7	**2a, 4a**
**6a**	3.44 (dd, 1H, *J* = 5.2, 5.8 Hz)	41.3	**8a**, **5a**, **4a**
**NH**	8.26 (t, 1H, *J* = 5.8, N**H**COCH_3_)	–	**8a**, **6a**
**8a**	–	170.0	–
**9a**	1.85 (s, 3H, CH_3_)	22.4 (1.9)	**8a**
**1b**	–	139.2 (d, *J*_*CF*_ = 11.0 Hz)	–
**2b**	7.60 (dd, 1H, *J* = 13.5, 2.3 Hz)	105.5 (d, *J*_*CF*_ = 28.7 Hz)	**6b**, **4b, 3b, 1b**
**3b**	–	158.9 (d, *J*_*CF*_ = 244.5 Hz)	–
**4b**	–	122.4 (d, *J*_*CF*_ = 13.4 Hz)	–
**5b**	7.56 (dd, 1H, *J* = 8.3, 9.2 Hz)	130.7 (d, *J*_*CF*_ = 4.7 Hz)	**3b**, **1b**, **1c**
**6b**	7.42 (dd, 1H, *J* = 8.6, 2.3 Hz)	113.9 (d, *J*_*CF*_ = 2.9 Hz)	**2b**, **4b, 5b(w)**
**1c**	–	133.5	–
**2c/6c**	7.50 (dd, 2H, *J* = 8.3, 1.5 Hz, Ar–**H**)	128.6	**4c, 2c/6c, 4b**
**3c/5c**	7.24 (d, 2H, *J* = 8.3 Hz, Ar–**H**)	127.5	**1c, 3c/5c, 7c**
**4c**	–	137.1	–
**7c**	4.34 (bs, 2H, N-**CH**_**2**_-Ar)	49.2	No
**6d**	4.49 (bs, 2H, triazole-**CH**_**2**_-N-)	38.8^b^	No
**5d**	7.56^b^ (bs, 1H, triazole)	133.0^b^	No
**4d**	–	No	–
**7e**	5.51 (bs, 2H, Ar–C**H**_**2**_)	50.1	**2e/6e, 1e**
**1e**	–	127.5	–
**2e/6e**	7.09 (bd, 2H, *J* = 8.5 Hz, Ar–**H**)	128.6	**4e, 2e/6e, 7e(w)**
**3e/5e**	6.90 (d, 2H, *J* = 8.7 Hz, Ar–**H**)	114.0	**1e, 3e/5e, 4e**
**4e**	–	158.9	–
O**CH**_**3**_	3.71 (s, 3H, Ar–OC**H**_**3**_)	55.0	**4e**
COOC(**CH**_**3**_ **)**_**3**_	1.35 (bs, 9H, COOC(**CH**_**3**_ **)**_**3**_)	27.7	–
COO**C**(CH_3_)_3_	–	79.9	–

^a^ *s* singlet, *bs* broad singlet, *d* doublet, *dd* doublet of doublets, *bd* broad doublet, *t* triplet, *m* multiplet, *w* weak, *no* not observed

^b^Spectra recorded at 353 K

^c^This column gives the carbon atoms showing correlation with a given proton

*Step I-2* NMR analysis: Based on the analysis of ^1^H and ^13^C chemical shifts, coupling patterns and the information obtained from 2D NMR experiments the structure of **2** could be proved (Table 2). The ^1^H{^13^C} HSQC and ^1^H{^13^C} gHMBC spectrum of reaction mixture of Step I-2 are presented in Additional file 1. The presence in the ^1^H{^13^C} gHMBC spectrum the peak correlation at 5.46 ppm/121.5 ppm between the signal of H7 protons (C**H**_**2**_ group) and a signal of protonated carbon atom **C**H assigned to the triazole ring as well as no occurrence of the peak correlation at 5.46 ppm/149.7 ppm between the signal of H7 protons (C**H**_**2**_ group) and unprotonated carbon atom signal assigned to the triazole ring suggests that one regioisomer, in which the triazole ring is substituted in position C4 was mainly obtained from this step. This conclusion is also confirmed by the occurring in this spectrum the peak correlation at 7.86 ppm/52.1 ppm between the proton signal assigned to triazole ring (C**H** group) and a carbon atom signal of **C**H_2_ group at position C7. The other regioisomer (substituted in position C5) was also formed in trace amount although, in contrast to patents [4, 5, 7, 8], where two regioisomers were formed in ratio 1.2:1. The difference may be results in using substrates and solvents of distinctive purity.

*Step I-3* ^1^H NMR (500 MHz, DMSO-d_6_) δ_H_ 1.32, 1.35, 1.39 (three br. s, 9H, COOC(C**H**_**3**_ **)**_**3**_); 3.72–3.73 (three br. s, 3H, Ar–OC**H**_**3**_); 4.20–4.50 (m, 4H, C**H**_**2**_–N–C**H**_**2**_); 5.48–5.51 (two br. s, 2H, Ar–C**H**_**2**_), 6.88–8.06 (m, 9H, Ar–**H** and triazole-C**H**).

*NMR analysis* In the ^1^H NMR spectrum of the studied sample, the signals were broadened or several signals occurred in a very narrow range of chemical shifts. Analysis of the correlation spectrum ^1^H{^13^C} HSQC was conducted, and confirmed the formation of compound **3.** In the aliphatic range of the ^1^H{^13^C}-HSQC spectrum, there were four correlation peaks of C**H**_**2**_ groups, which could be assigned to substrates or reaction products. Two peaks at 5.48 ppm/52.2 ppm and 5.49 ppm/50.1 ppm occurred in a similar region as C**H**_**2**_ group at position 7 of compound **2** and may be assigned to the same group in compound **3**. The presence of two other correlation peaks of the C**H**_**2**_ groups at 4.28–4.37 ppm/49.6 ppm and 4.29–4.38 ppm/41.3 ppm, which may be assigned to the fragment: triazol**–**C**H**_**2**_ **–**N(BOC)**–**C**H**_**2**_ **–**Ar of compound **3** together with the absence of the correlation peak that is characteristic of the C**H**_**2**_–NH_2_ of compound **2** at 3.71 ppm/37.1 ppm, unambiguously confirmed the expected course of the reaction.

*Step II-2* The analysis of chemical shifts (^1^H and ^13^C NMR) and multiplicity together with values of coupling constants (proton–proton, proton–fluorine, carbon–fluorine) confirm the present of *m*-substituted benzene ring (one of the substituents is fluorine atom) and 5-hydroxymethyl-2-oxazolidinone in the structure of compound **5.** The signal of quaternary carbon atom which was assigned to the benzene ring (C1′) is located at 140.1 ppm what indicates on its connecting with heteroatom–nitrogen. It can be conclude that benzene ring is attached to oxazolidinone via nitrogen atom (Table 3).

*Step II-6* Based on the analysis of chemical shifts, coupling patterns and the information obtained from 2D NMR experiments which allowed us to confirm the protons and carbon atoms connecting scheme, the structure of **9** could be proved (Table 4).

In particular, the occurrence of the AMX proton system additionally coupling with fluorine atom in aromatic part of ^1^H NMR spectrum and six signals in ^13^C NMR spectrum, which show coupling with the fluorine atom between 74.0 and 161.1 ppm (three signals of the quaternary carbon atom and three signals of CH groups) confirm the presence of the tri-substituted benzene ring in the structures of the compound **9**. Additionally very characteristic chemical shift of signal which was assigned to quaternary carbon atom at 74.0 ppm confirm, that benzene ring is substituted also by iodine atom (fluorine and iodine atoms are substituted at positions 3′ and 4′, respectively).

Comparing the aliphatic region of ^1^H NMR spectra of compounds **9** and **5** indicates a very high similarity. In spectrum of **9** there is no proton signal of O**H** group at 5.23 ppm while the signal of CH_3_ group at 1.83 ppm appear. Additionally it is observed N**H** group signal at 8.22 ppm. Comparing the aliphatic region of ^13^C NMR spectra of compound **9** and **5** also shows slight differences. In spectrum of **9** there is not observed carbon atom signal of CH_2_ group at 61.5 ppm characteristic for compounds **5** while there are signals of CH_2_ group at 41.2 ppm and signal of CH_3_ group at 22.3 ppm. Additionally signal of quaternary carbon atom at 169.9 ppm is observed. The changes observed in the NMR spectra of compound **9** compared to the spectra of compound **5** indicate that the 2-oxazolidinone is substituted at the 5-position by the methylacetamide group in compound **9.**

*Step I-4*, the most essential step. NMR analysis: Comparison of the structures of compound **10** and substrates **3** and **9** led to the conclusion that neglected changes between the chemical shifts of most proton signals in the ^1^H NMR spectrum of **10** versus to the chemical shifts of proton signals in the spectra of compounds **3** and **9** should be anticipated. Significant changes in the chemical shift can be expected only for the signals of protons of aromatic rings in substrates **3** and **9**, between which the bond is formed. Actually, following chemical shifts, changes were observed: 0.27 ppm upfield, 0.23 ppm downfield, and 0.24 ppm upfield, for proton signals H5b, H6b and H2c/H6c, respectively. The analysis of the carbon and 2D correlation spectra were additional proof of the formation of compound **10**. Moreover, in the ^13^C NMR spectrum of studied sample signals of the quaternary carbon atom C4b of an aromatic ring substituted by iodine atom at 74.0 ppm (d, *J*_CF_ = 21.2 Hz) characteristic for compound **9** was not observed, while other signals of the quaternary carbon atom at 122.4 ppm, which could be assigned to the carbon atom C4b of **10**, was noticed. This signal is a doublet with a coupling constant *J*_CF_ = 13.4 Hz, which confirms proper assignment to the carbon atom of an aromatic ring substituted by fluorine. Furthermore, the presence in the ^1^H{^13^C} gHMBC spectrum (attached in Additional file 1) peaks correlation at 7.50 ppm/122.4 ppm between the signal of aromatic protons which was assigned to H2c/H6c protons and the above-mentioned signal of quaternary carbon atom assigned to C4b position and at 7.56 ppm/133.5 ppm between the signal of aromatic protons assigned to H5b proton and a quaternary carbon atom assigned to C1c position immediately demonstrated the direct bond between two aromatic rings of substrates, and consequently the formation of compound **10** (Table 5).

**Fig. 3:**
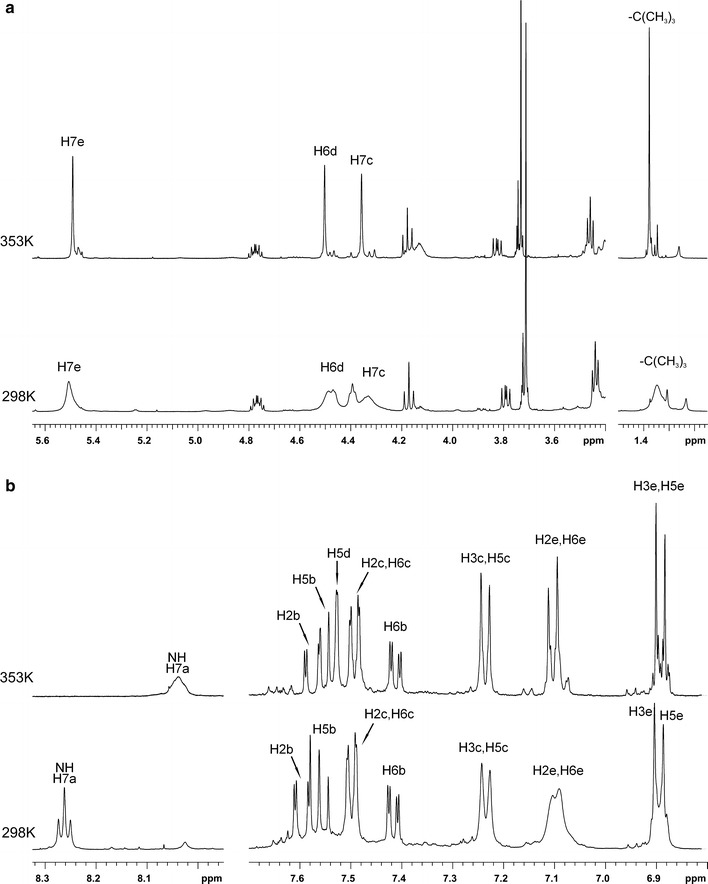
**a** The aliphatic region of the ^1^H NMR spectra of compound **10** recorded at 298 K (*down*) and 353 K (*up*); **b** the aromatic region of the ^1^H NMR spectra of compound **10** recorded at 298 K (*down*) and 353 K (*up*)

In the ^1^H NMR spectrum of compound **10**, recorded at 298 K on a 500 MHz spectrometer, some proton signals showed broadening, most probably due to hindered rotation along bonds N-BOC. The large broadening was observed for signals located at 7.09 ppm, 5.51 ppm, in the range of 4.3–4.5 ppm and 1.31–1.38 ppm, which were assigned to aromatic protons H2e/H6e, protons of C**H**_2_ group H7e, and protons of the C**H**_2_ groups from fragment C**H**_2_(7c)–N–C**H**_2_(6d), as well to the tert-butyl group protons, respectively (Fig. 3a). The resonance of triazole ring proton was not present in the ^1^H NMR spectrum due to the extremely large broadening (Fig. 3b). However, in the ^1^H NMR spectrum recorded at 353 K, the aforementioned signals significantly sharpened; e.g. the signal of a triazole ring proton appeared as a broad singlet in the expected range at 7.56 ppm (Fig. 3b), and the signals of the C**H**_2_ groups of fragments C**H**_2_(7c)–N–C**H**_2_(6d) were narrow singlets at 4.34 and 4.49 ppm, respectively (Fig. 3a).

In the ^1^H{^13^C} gHMBC spectrum recorded at 298 K heteronuclear peaks, a correlation between the proton signals of the C**H**_2_ groups of the fragment C**H**_2_ (7c)–N–C**H**_2_ (6d) and a proton signal of triazole ring to the appropriate signals of carbon atoms two or three bonds away was not observed, which was caused by a very large broadening of the proton signals. Also, in the ^13^C NMR spectrum registered at 298 K, signals of the triazole ring carbon atoms C5d and C4d were broadened in such an extent that in practice they were not observed.

**Fig. 4:**
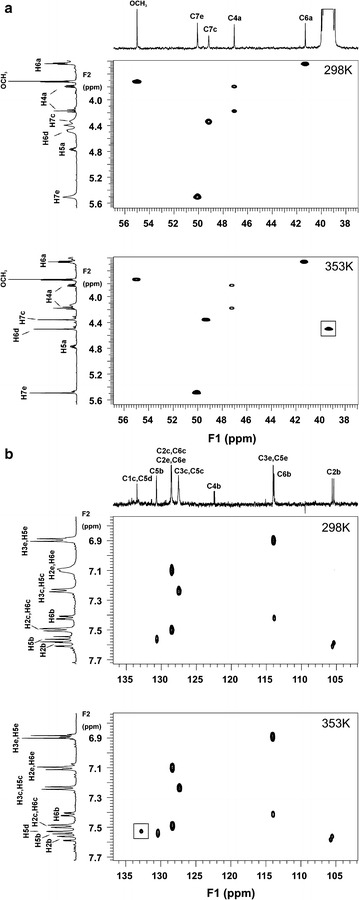
**a** The aliphatic region of the ^1^H{^13^C} HSQCAD spectra of compound **10** recorded at 298 K (*up*) and 353 K (*down*); **b** the aromatic region of the ^1^H{^13^C} HSQCAD spectra of compound **10** recorded at 298 K (*up*) and 353 K (*down*). Correlation peaks for **CH**(5d) and **CH**_**2**_(6d) groups were observed only in spectrum recorded at 353 K (crosspeak marked in *rectangle*)

Heteronuclear correlation peaks (along single H–C bond) for C**H**(5d) and **C**H_2_(6d) groups were observed in the ^1^H{^13^C} HSQC spectrum recorded at 353 K but not in the spectrum recorded at 298 K (Fig. 4—cross-peak marked in a rectangle).

## Conclusions

Proposed changes to the synthesis of RAD [5–7] have given positive results; the improvement of overall yield increased from 47.3 to 54.5%. The synthesis route was controlled by means of NMR, IR, Raman and ECD methods. The intermediate products to the final product were characterised and proved their structure, specifying chirality and purity, respectively. Complementary FT-IR and Raman techniques supported by a theoretical approach allowed the detailed analysis of symmetric and asymmetric vibrations in the tested products. The information obtained from 2D NMR experiments concerning the proton and carbon connecting scheme in the studied compounds has enabled their structures to be confirmed and finally proven. The ECD method was especially valuable for the analysis of chiral purity of radezolid.

Theoretical modelling with the application of a QZVP basis set has allowed accurate optimisation of the geometry of the intermediate products; the results revealed good agreement with the experimental outcome.

## Experimental section

### Chemistry

The following materials and reagents were used for synthesis: silica gel 60 (70–230 mesh) was purchased from Merck GmbH (Darmstadt, Germany), 3-fluoroaniline, (*R*)-glycidyl butyrate, *n*-buthyllithium (1.6 M in hexane), sodium azide, triethylamine, methanesulphonyl chloride, trifluoroacetic acid silver salt, thioacetic acid, iodine, tetrakis(triphenylphosphine)palladium(0), 4N hydrogen chloride in 1,4-dioxane and trifluoroacetic acid, propargylamine, 4-formylphenylboronic acid, sodium triacetoxyborohydride, and di-tert-butyl dicarbonate were purchased from Sigma-Aldrich (Steinheim, Germany), 4-methoxybenzyl chloride, benzyl chlorformate were purchased from TCI (Tokyo, Japan), sodium bicarbonate, magnesium sulphate, sodium sulphate, potassium carbonate, sodium chloride, ammonium chloride, hexane, ethyl acetate, methanol, acetone, tetrahydrofuran, hydrochloric acid, ethanol, toluene, dichloromethane, and chloroform were purchased from POCH (Gliwice, Poland) and all were of analytical grade. *N*,*N*-dimethylformamide, and acetonitrile were purchased from Rathburn Chemicals (Walkerburn, Great Britain) and were of HPLC grade.

The reference standard of RAD was purchased from ApexBio Technology LLC (Houston, TX, USA).

### Instrumentation

#### Nuclear magnetic resonance (NMR)

The NMR spectra were recorded at 298 and 353 K on a Varian VNMRS-500 spectrometer operated at 499.8 and 125.7 MHz for ^1^H and ^13^C, respectively. The spectrometer was equipped with an inverse ^1^H{^13^C/^15^N} 5 mm PFG Triple Resonance Probe with an actively shielded z-gradient coil. The NMR experiments were run by using the standard Varian software. An amount of compound or reaction mixture of 5–10 mg was dissolved in 0.7 mL of DMSO-d_6_ and transferred to a 5 mm NMR tube. The ^1^H and ^13^C chemical shifts, δ, given in ppm, were referenced against solvent DMSO-d_6_ (2.504 ppm for ^1^H and 39.4 ppm for ^13^C).

##### ^1^H NMR

A standard single-pulse experiment was used to acquire the ^1^H spectrum using a 6000 Hz spectral window, 30° pulse width, an acquisition time of 3.0 s, with 64 K complex data points. The FIDs were processed with zero-filling.

##### ^13^C NMR

The ^13^C NMR spectra were run by using a spectral range of 32 kHz, 30° pulse width, acquisition time of 1.0 s, a relaxation delay of 0.5 s, and by collecting 32 K complex data points.

##### ^1^H{^13^C} HSQCAD

The phase sensitive adiabatic HSQC (*H*eteronuclear *S*ingle *Q*uantum *C*orrelation) [24–26] with multiplicity editing spectra were acquired with an acquisition time of 0.2 s, relaxation delay of 1.0 s and spectral windows of 4500–6000 Hz in F2 and 20,100–21,100 Hz in F1. 512 complex data points were collected in the indirectly detected dimension (^13^C) with 2–8 scans and 2048 points per increment. The data were linearly predicted to 1 K and zero filled to 4 K complex data points in F1 and processed using the cosine window function in both dimensions prior to Fourier transformation. The proton and carbon π/2 pulse lengths were 6.7–7.0 and 14.8 μs, respectively.

##### ^1^H{^13^C} gHMBCAD

The phase-sensitive gradient-selected adiabatic HMBC (*H*eteronuclear *M*ultiple *B*ond *C*orrelation) [27, 28] spectra were acquired over a sweep width of 4500–6000 Hz in F2 and 23,800–26,400 Hz in F1, with an acquisition time of 0.2 s, relaxation delay of 1.0 s and ^n^J_(C,H)_ = 8 Hz. Overall, 512 complex data points were collected in the indirectly detected dimension (^13^C) with 4–128 scans and 2048 points per increment. The data were linearly predicted to 1 K and zero filled to 4 K complex data points in F1 and processed using sine-bell square multiplication in F2 and Gaussian window function in F1 dimensions prior to Fourier transformation.

#### Vibrational spectroscopy

The vibrational infrared spectra of RAD were recorded between 4000 and 100 cm^−1^ in powder, at room temperature, with a Bruker Equinox 55 FT-IR spectrometer equipped with a Bruker Hyperion 1000 microscope.

Raman scattering spectra were obtained with a LabRAM HR800 spectrometer (HORIBA JobinYvon) with laser excitation λexc = 633 nm (He–Ne laser). In each case, the power of the laser beam focused on the sample was less than 1 mW to avoid damage to the sample.

#### Chiroptical method

Circular dichroism of RAD spectra was established using a Jasco J-715 spectrometer.

#### Computation details

All calculations were performed using Gaussian 09 software and visualised using GaussView [29]. The harmonic vibrational frequencies for spectroscopic analysis (for FT-IR and Raman spectra) were carried out with DFT using a B3LYP hybrid functional with a Quadruple Zeta Valence plus Polarisation function (QZVP) basis set. The NMR magnetic shielding and spin–spin coupling constants were calculated using the gauge-independent atomic orbital (GIAO) method under DFT [30]. The B3LYP functional employing the standard 6–311++G(d, p) basis set was used. The polarizable continuum model (PCM) using the standard integral equation formalism variant (IEFPCM) was used to simulate the influence of water as a solvent [31]. The GIAO calculations were preceded by precise searching for the lowest energy conformers conducted at the same level of theory.

## Additional file

**Additional file 1: Figure SI** a) The calculated (black) and experimental (red) FT-IR absorption spectra in room temperature of the 5; b) The calculated (black) and experimental (red) Raman scattering spectra in room temperature of the 5. Figure S2. a) The calculated (black) and experimental (blue) FT-IR absorption spectra in room temperature of the 9; b) The calculated (black) and experimental (blue) Raman scattering spectra in room temperature of the 9. Figure S3. a) The experimental FT-IR absorption spectra in room temperature of the 9 (blue), 5 (red), 3 (black), and RAD (green); b) The experimental Raman scattering spectra in room temperature of the 9 (blue), 5 (red), 3 (black), and RAD (green). Figure S4. a) The experimental FT-IR absorption spectra in room temperature of the 3; b) The experimental Raman scattering spectra in room temperature of 3. Figure S5. The 1H{13C} HSQC spectrum of “reaction mixture of Step I-2” recorded at 298 K. The cross peaks of CH, CH2 and CH3 groups marked in red belong to compound 2. The cross peaks marked in black probably belong both to another regioisomer of compound 2 substituted in position C5 or to unidentified by-products. The above-mentioned compouds account for about 20% of the tested sample. Figure S6. a) The aromatic part of 1H{13C} gHMBC spectrum of “reaction mixture of Step I-2” recorded at 298 K. b) The aliphatic part of 1H{13C} gHMBC spectrum of “reaction mixture of Step I-2” recorded at 298 K. Figure S7. The part of the 1H{13C} gHMBC spectrum of compound 10 recorded at 298 K (cross peaks marked in red) demonstrated the direct bond between two aromatic rings of substrates 3 and 9 and consequently the formation of compound 10.
