# Supplementary material for: Comprehensive spectral identification of key intermediates to the final product of the chiral pool synthesis of radezolid
Source: Chem Cent J. 2017 Aug 9;11:82. doi: 10.1186/s13065-017-0309-x (PMC5549672; doi:10.1186/s13065-017-0309-x)
Supplement: Supplementary file 1 — Additional file 1: Figure SI a) The calculated (black) and experimental (red) FT-IR absorption spectra in room temperature of the 5; b) The calculated (black) and experimental (red) Raman scattering spectra in room temperature of the 5. Figure S2. a) The calculated (black) and experimental (blue) FT-IR absorption spectra in room temperature of the 9; b) The calculated (black) and experimental (blue) Raman scattering spectra in room temperature of the 9. Figure S3. a) The experimental FT-IR absorption spectra in room temperature of the 9 (blue), 5 (red), 3 (black), and RAD (green); b) The experimental Raman scattering spectra in room temperature of the 9 (blue), 5 (red), 3 (black), and RAD (green). Figure S4. a) The experimental FT-IR absorption spectra in room temperature of the 3; b) The experimental Raman scattering spectra in room temperature of 3. Figure S5. The 1H{13C} HSQC spectrum of “reaction mixture of Step I-2” recorded at 298 K. The cross peaks of CH, CH2 and CH3 groups marked in red belong to compound 2. The cross peaks marked in black probably belong both to another regioisomer of compound 2 substituted in position C5 or to unidentified by-products. The above-mentioned compouds account for about 20% of the tested sample. Figure S6. a) The aromatic part of 1H{13C} gHMBC spectrum of “reaction mixture of Step I-2” recorded at 298 K. b) The aliphatic part of 1H{13C} gHMBC spectrum of “reaction mixture of Step I-2” recorded at 298 K. Figure S7. The part of the 1H{13C} gHMBC spectrum of compound 10 recorded at 298 K (cross peaks marked in red) demonstrated the direct bond between two aromatic rings of substrates 3 and 9 and consequently the formation of compound 10. [file 13065_2017_309_MOESM1_ESM.docx]

**Supplementary information to:**

**Comprehensive Spectral Identification of Key Intermediates to the Final Product of the Chiral Pool Synthesis of Radezolid**

Katarzyna Michalska^1*^, Elżbieta Bednarek^2*^, Ewa Gruba^1^,

Kornelia Lewandowska^3^, Mikołaj Mizera^4^, Judyta Cielecka-Piontek^4*^

*^1^ Department of Antibiotics and Microbiology, National Medicines Institute, Chelmska 30/34, 00-725 Warsaw, Poland, E-mail: k.michalska@nil.gov.pl*

*^2^ Department of Counterfeit Medicinal Products and Drugs, National Medicines Institute, Chelmska 30/34, 00-725 Warsaw, Poland, E-mail: e.bednarek@nil.gov.pl*

*^3^ Department of Molecular Crystals, Institute of Molecular Physics of the Polish Academy of Sciences, Smoluchowskiego 17, 60-179 Poznan, Poland*

*^4^ Department of Pharmaceutical Chemistry, Poznan University of Medical Sciences, Grunwaldzka 6, 60-780 Poznan, Poland, E-mail: judyta.piontek@ump.edu.pl*

*Synthesis*

Synthesis of RAD **N-{[(5*S*)-3-[3-fluoro-4-(4-{[(1H-1,2,3-triazol-5-ylmethyl)amino]methyl}phenyl)phenyl]-2-oxo-1,3-oxazolidin-5-yl]methyl}acetamide** (**12**) was obtained according to the procedures depicted in Scheme 1, as described in detail below.

Step I-1. Preparation of **4-methoxybenzyl azide** (**1**).

To 4-methoxybenzyl chloride (0.56 g, 3.56 mmol) in anhydrous DMF (2 mL), solid sodium azide was added (0.23 g, 3.55 mmol) at room temperature. The reaction was stirred for 24 h. Then, water (H_2_O) was added to the reaction mixture and extracted with ethyl acetate (EtOAc). The organic layer was washed with brine and dried over anhydrous MgSO_4_, filtered and concentrated *in vacuo* to obtain the title compound **1** as a colourless oil (0.55 g, 94% yield).

Step I-2. Preparation of ***C*-[1-(4-methoxybenzyl)-1*H*-[1,2,3]triazol-4-yl]methylamine** and ***C*-[3-(4-methoxybenzyl)-3*H*-[1,2,3]triazol-4-yl]methylamine** (**2**).

A solution of 4-methoxybenzyl azide **1** (0.33 g, 2.00 mmol) in toluene (5 mL) was treated with propargylamine (0.16 g, 0.21 mL, 3.00 mmol, 1.5 equiv.) at 25 °C, then the resulting reaction mixture was warmed up to gentle reflux at 105-110 °C for 21 h. Next, the reaction mixture was cooled down to room temperature before being concentrated *in vacuo*. The oily residue was then treated with 30% EtOAc-hexane (20 mL), and the resulting mixture was warmed to reflux and stirred at reflux for 30 min; after cooling down to room temperature, the reaction was stirred for 1 h. The pale-yellow solid of **2** was then collected by filtration, washed with EtOAc-hexane (2×10 mL), and dried *in vacuo* overnight to afford the crude, cycloaddition product (0.42 g, 95%) (Table 2).

Step I-3. Preparation of **4-({*t*-butoxycarbonyl-[1-(4-methoxybenzyl)-1*H*-[1,2,3]triazol-4-ylmethyl]amino}methyl)phenylboronic acid** (**3**).

To a solution of the mixture of **2** (0.22g, 1.03 mmol) in tetrahydrofuran (5 mL), 4-formylphenylboronic acid (0.15 g, 0.98 mmol, 0.95 equiv.) was added at room temperature, and the resulting reaction mixture was stirred at room temperature for 10 min. Then, sodium triacetoxyborohydride (NaB(OAc)_3_H, 0.33 g, 1.55 mmol, 1.5 equiv.) was added in three portions over the period of 1.5 h at room temperature, and the resulting reaction mixture was stirred at room temperature for an additional 4 h. After that period of time, the reaction was treated with H_2_O (15 mL) and heated with solid potassium carbonate (0.43 g, 3.10 mmol, 3.0 equiv.) and di-tert-butyl dicarbonate (0.23 g, 1.03 mmol, 1.0 equiv.) at room temperature; the reaction mixture was stirred for 5 h. The entire mixture was transferred to a separatory funnel with the aid of EtOAc (15 mL) and H_2_O (10 mL) and the mixture was extracted. The combined organic extracts were washed with H_2_O (5 mL), 1.5 N aqueous HCl solution (2×10 mL), H_2_O (10 mL), and saturated aqueous NaCl solution (10 mL), dried over MgSO_4_, and concentrated *in vacuo*. The crude 4-({tert-butoxycarbonyl-[1-(4-methoxy-benzyl)-1*H*-[1,2,3]triazol-4-ylmethyl]-amino}-methyl)-phenylboronic acid **3** (0.43 g, 96%) was obtained as a pale-yellow oil, which solidified upon standing at room temperature.

The synthesis procedure for ***N*-{[(5*S*)-3-(3-fluoro-4-iodophenyl)-2-oxo-1,3-oxazoilidin-5-yl]methyl}acetamide** (**9**) is well known and published in many papers (Scheme 2) [4,5,B].

Step II-1. Preparation of ***N*-carboxyloxy-3-fluoroaniline** (**4**).

To a solution of 3-fluoroaniline (5 g, 45 mmol) and NaHCO_3_ (7.5 g, 90 mmol) in THF (50 mL) at 0°C, benzyl chlorformate (7.7 mL, 55 mmol) was slowly added. After stirring for 2.5 h at 0 °C, H_2_O (10 mL) was added to the mixture, warmed up to room temperature and extracted with EtOAc. The organic layer was washed with brine, dried over MgSO_4_, filtered and concentrated *in vacuo*. The residue was washed and crystallised from n-hexane to obtain the title compound as light pink crystals which became off-white after being left in air overnight (6.4 g, 58%).

Step II-2. Preparation of **(5*R*)-3-(3-fluorophenyl)-5-hydroxymethyl-2-oxooxazolidine** (**5**).

A solution of **4** (2 g, 8.15 mmol) in dry THF (20 mL) was cooled with dry ice/acetone bath to -78°C and then n-buthyllithium (5.6 mL of a 1.6 M solution in hexane, 9 mmol) was added under nitrogen conditions. (*R*)-glycidyl butyrate (1.272 mL, 1.294 g, 9 mmol) was added dropwise *via* a syringe and the cooling bath was left for 2 h and then allowed to dissipate overnight, with the reaction mixture reaching ambient temperature. The reaction mixture was quenched by the careful addition of saturated aqueous ammonium chloride. The entire mixture was transferred to a separatory funnel with the aid of dichloromethane washings the mixture was extracted. The combined organic extracts were dried over Na_2_SO_4_, filtered and concentrated *in vacuo* to give an pale yellow oil which was purified by chromatography over silica gel, eluting with 10% acetonitrile (CH_3_CN) in chloroform (CHCl_3_) containing 1% methanol, to afford a white solid (1.1763 g, 91%) (Table 3).

Step II-3. Preparation of **(5*R*)-3-(3-fluoro-4-iodophenyl)-5-hydroxymethyloxazolidin-2-one** (**6**).

A solution of **5** (0.9 g, 4.2 mmol) in a CH_3_CN (10 mL) was stirred for 10 min and then trifluoroacetic acid silver salt (1.25 g, 5.7 mmol) and iodine crystals (1.17 g, 4.6 mmol) were added. The solution was stirred for 24 h at room temperature. To the reaction mixture, H_2_O was added and extracted with EtOAc. The organic layer was washed with brine and dried over anhydrous MgSO_4_, filtered and concentrated *in vacuo* to obtain the title compound as a yellow solid (1.27 g, 90%).

Step II-4. Preparation of **[(5*R*)-3-(3-fluoro-4-iodophenyl)-2-oxo-1,3-oxazolidin-5-yl]methyl methanesulfonate** (**7**).

(5*R*)-3-(3-fluoro-4-iodophenyl)-5-hydroxymethyloxazolidin-2-one **6** (1.00 g, 2.97 mmol) was dissolved in dichloromethane (10 mL) and cooled to 0 °C. Triethylamine (0.44 g, 4.36 mmol) and methanesulphonyl chloride (0.45 g, 3.57 mmol) were added and the reaction was stirred for 18 h, slowly warming up to room temperature. The solution was diluted with a saturated solution of sodium bicarbonate and extracted using dichloromethane (3 x 10 mL). The organic layer was dried over MgSO_4_, filtered and concentrated *in vacuo* to obtain the title compound as a pale yellow solid (1.20 g, 97%).

Step II-5. Preparation of **(5*R*)-5-(azidomethyl)-3-(3-fluoro-4-iodophenyl)-1,3-oxazolidin-2-one** (**8**).

[(5*R*)-3-(3-fluoro-4-iodophenyl)-2-oxo-1,3-oxazolidin-5-yl]methyl methanesulfonate **7** (1.1 g, 2.63 mmol) was dissolved in N,N-dimethylformamide (10 mL). Sodium azide (0.36 g, 5.55 mmol) was added and the reaction was stirred at 75 °C for 20 h. The mixture was poured into saturated sodium bicarbonate and extracted with EtOAc. The organic layer was washed with H_2_O, dried over MgSO_4_, filtered and concentrated *in vacuo* to obtain the title compound as a yellow solid (0.76 g, 80%).

Step II-6. Preparation of ***N*-{[(5*S*)-3-(3-fluoro-4-iodophenyl)-2-oxo-1,3-oxazoilidin-5-yl]methyl}acetamide** (**9**).

(5*R*)-5-(azidomethyl)-3-(3-fluoro-4-iodophenyl)-1,3-oxazolidin-2-one **8** (0.70 g, 1.96 mmol) was suspended in thioacetic acid (1.5 mL) and the reaction was carried out under nitrogen at room temperature for 18 h. The resulting suspension was concentrated *in vacuo*, crystallised from MeOH/acetone and then purified by chromatography over silica gel, eluting with dichloromethane, to afford an off-white solid (0.48 g, 65%) (Table 4).

Step I-4. Preparation of **(5*S*)-{4’-(acetylaminomethyl)-2-oxo-oxazolidin-3-yl]-2’-fluorobiphenyl-4-ylmethyl}-[1-(4-methoxybenzyl)-1*H*-[1,2,3]triazol-4-ylmethyl]carbamic acid t-butyl ester** (**10**).

A suspension of the crude **3** (0.36 g, 0.81 mmol) and *N*-{[(5*S*)-3-(3-fluoro-4-iodophenyl)-2-oxo-1,3-oxazoilidin-5-yl]methyl}acetamide **9** (0.27 g, 0.73 mmol, 0.90 equiv) in toluene (5 mL) was treated with powdered K_2_CO_3_ (0.33 g, 2.42 mol, 3.0 equiv), EtOH (2 mL), and H_2_O (2 mL) at 25 °C; the resulting mixture was then degassed by a stream of nitrogen at 25 °C. Then Pd(PPh_3_)_4_ (84.1 mg, 0.07 mmol, 0.1 equiv) was subsequently added to the reaction mixture, and the resulting reaction mixture was degassed with nitrogen at 25 °C, then warmed up to gentle reflux for 22 h. The reaction mixture was cooled to room temperature, treated with H_2_O (10 mL) and EtOAc (10 mL). The two layers were then separated, and the aqueous layer was extracted with EtOAc (10 mL). The combined organic extracts were washed with H_2_O (2.5 mL), 1.5 N aqueous HCl solution (2×3 mL), H_2_O (5 mL), and saturated aqueous NaCl solution (10 mL), dried over MgSO_4_, and concentrated *in vacuo*. The residual oil was solidified upon standing at room temperature to afford the crude (5*S*)-{4’-[5-(acetylamino-methyl)-2-oxo-oxazolidin-3-yl]-2’-fluoro-biphenyl-4-ylmethyl}-[1-(4-methoxy-benzyl)-1*H*-[1,2,3]triazol-4-ylmethyl]-carbamic acid tert-butyl ester **10** (0.42 g, 86%) (Table 5).

Step I-5. Preparation of **(5*S*)-*N*-{3-[2-fluoro-4’-({[1-(4-methoxybenzyl)-1*H*-[1,2,3]triazol-4-ylmethyl]-amino}-methyl)-biphenyl-4-yl]-2-oxo-oxazolidin-5-ylmethyl}-acetamide hydrochloride** (**11**).

To a solution of **10** (0.37 g, 0.57 mmol) in EtOAc (3 mL) and MeOH (1 mL), a solution of 4 N hydrogen chloride in 1,4-dioxane (1.13 mL, 4.53 mmol, 8.0 equiv) was added at room temperature; the resulting reaction mixture was stirred at room temperature for 18 h. Then, the solvents were removed *in vacuo*. The residue was suspended in 10 mL 5% MeOH in acetonitrile (CH_3_CN), and the resulting slurry was stirred at room temperature for 1 h. The solids were then collected by filtration, washed with toluene (2×10 mL) and 5% MeOH in CH_3_CN (2×5 mL), and dried *in vacuo* to afford the crude, (5*S*)-*N*-{3-[2-fluoro-4’-({[1-(4-methoxy-benzyl)-1*H*-[1,2,3]triazol-4-ylmethyl]-amino}-methyl)-biphenyl-4-yl]-2-oxo-oxazolidin-5-ylmethyl}-acetamide hydrochloride **11** (0.30 g, 87% yield) as off-white crystals.

Step I-6. Preparation of **(5*S*)-*N*-[3-(2-fluoro-4'-{[(1*H*-[1,2,3]triazol-4-ylmethyl)-amino]-methyl}-biphenyl-4-yl)-2-oxo-oxazolidin-5-ylmethyl]-acetamide** (**12**).

A solution of the crude mixture of **11** (0.26 g, 0.44 mmol) in trifluoroacetic acid (4 mL) was warmed up to gentle reflux at 72-75 °C for 18 h. Then, the solvents were removed *in vacuo*. The residual solids were then treated with EtOAc (5 mL) H_2_O (7.5 mL) and acetone (2 mL) at room temperature. The resulting mixture was stirred at room temperature for 1h before the solids were collected by filtration, washed with EtOAc (2×5 mL) and H_2_O (2×5 mL), and dried *in vacuo* to afford (5*S*)-*N*-[3-(2-fluoro-4′-{[(1*H*-[1,2,3]triazol-4-ylmethyl)-amino]-methyl)-biphenyl-4-yl)-2-oxo-oxazolidin-5-ylmethyl]-acetamide **12** as an off-white powder. To the filtrate, hexane (3 mL) was added; the mixture was stirred at room temperature for 30 min and then two layers were separated. To the aqueous layer, acetone (2 mL) was added and then a new portion of crystals of **12** was collected. The overall yield is 0.17 g, 85% (Table enclosed below).


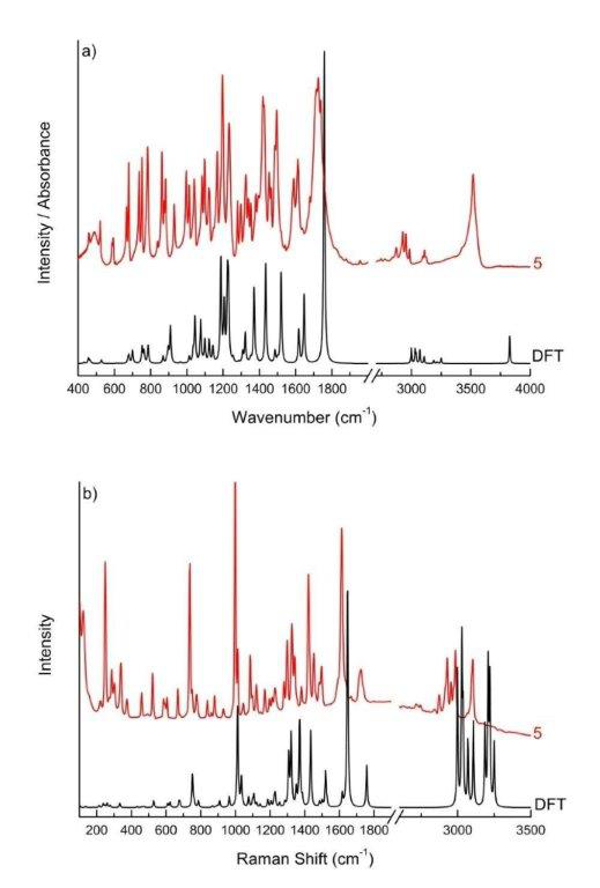


Fig. 1-SI. a) The calculated (black) and experimental (red) FT-IR absorption spectra in room temperature of the **5**;

b) The calculated (black) and experimental (red) Raman scattering spectra in room temperature of the **5**.


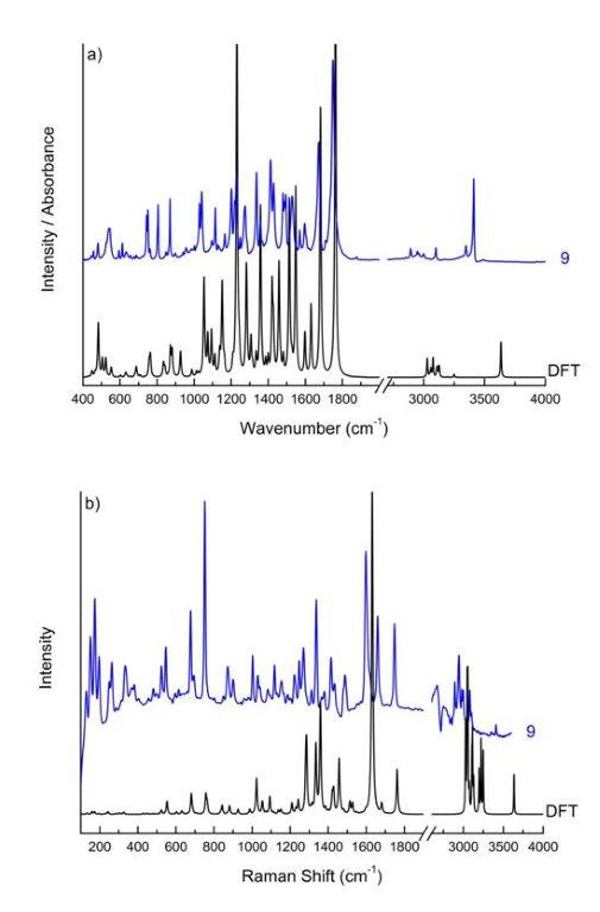


Fig. 2-SI. a) The calculated (black) and experimental (blue) FT-IR absorption spectra in room temperature of the **9**;

b) The calculated (black) and experimental (blue) Raman scattering spectra in room temperature of the **9**.


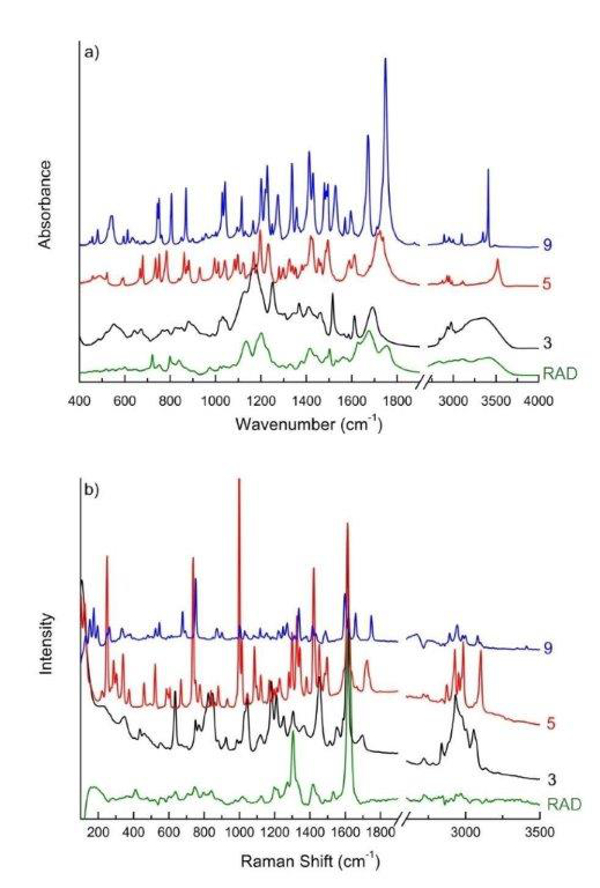


Fig. 3-SI. a) The experimental FT-IR absorption spectra in room temperature of the **9** (blue), **5** (red), **3** (black), and **RAD** (green);

b) The experimental Raman scattering spectra in room temperature of the **9** (blue), **5** (red), **3** (black), and **RAD** (green).


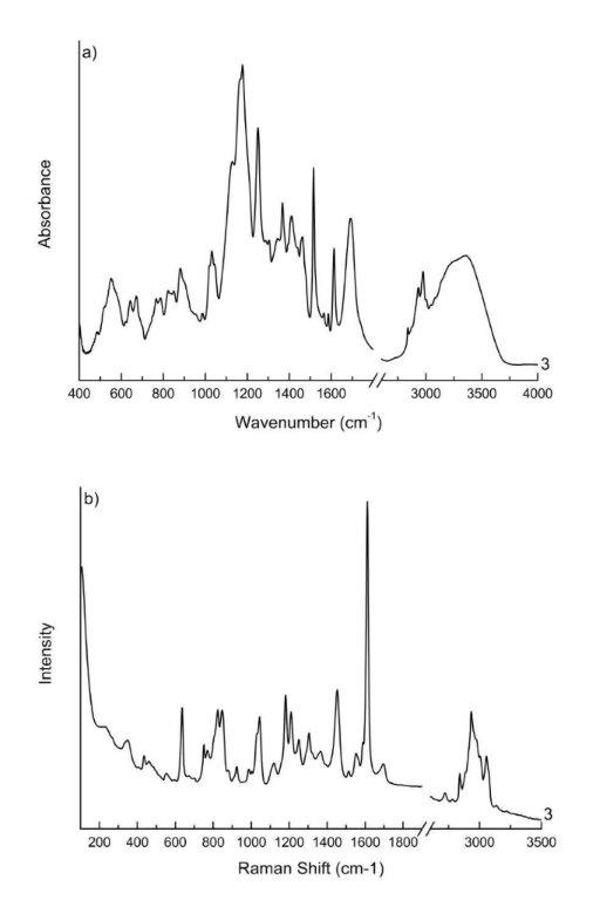


Fig. 4-SI. a) The experimental FT-IR absorption spectra in room temperature of the **3**;

b) The experimental Raman scattering spectra in room temperature of **3**.


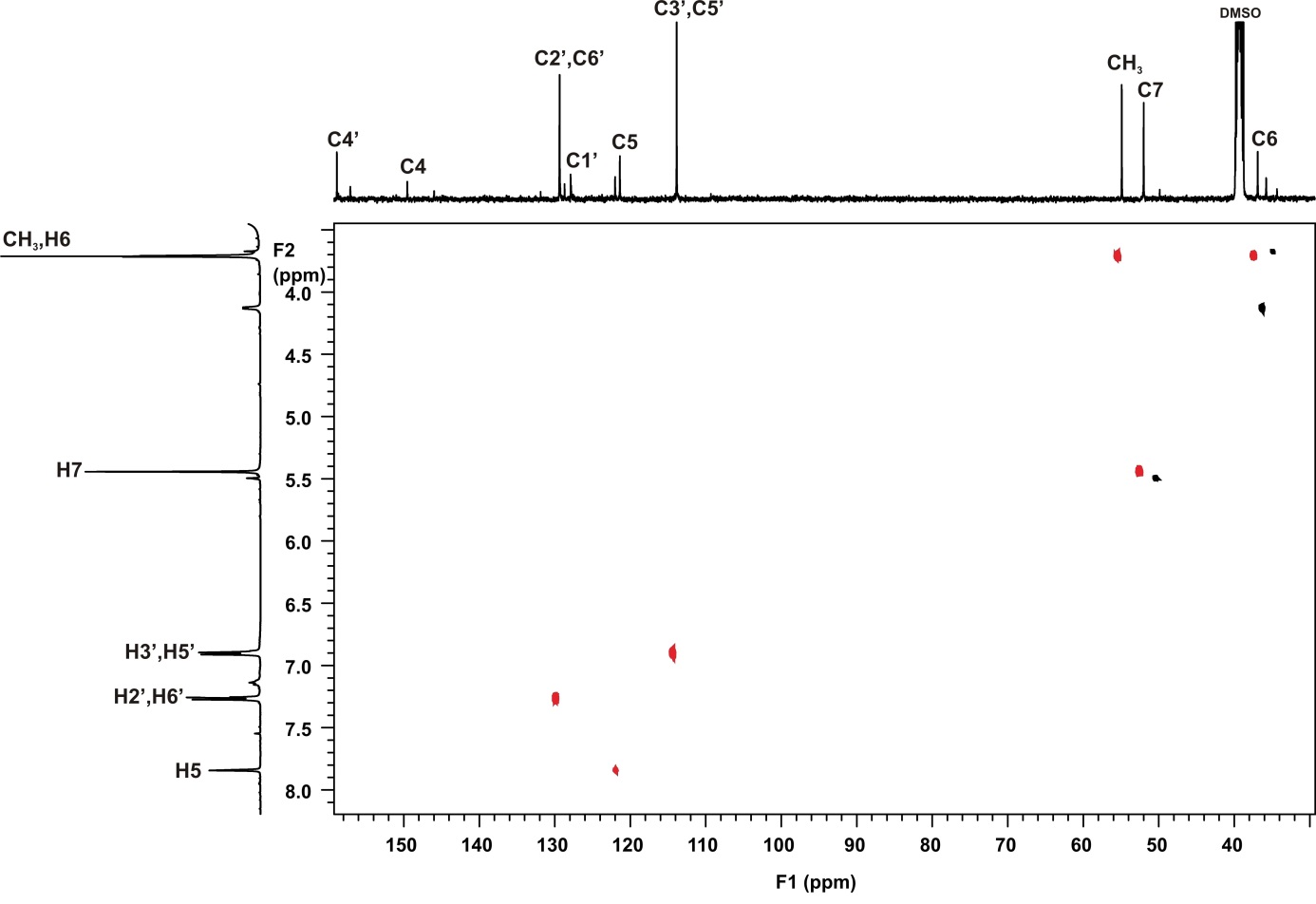


Fig. 5-SI. The ^1^H{^13^C} HSQC spectrum of “reaction mixture of Step I-2” recorded at 298 K. The cross peaks of CH, CH_2_ and CH_3_ groups marked in red belong to compound **2**. The cross peaks marked in black probably belong both to another regioisomer of compound **2** substituted in position C5 or to unidentified by-products. The above-mentioned compouds account for about 20% of the tested sample.


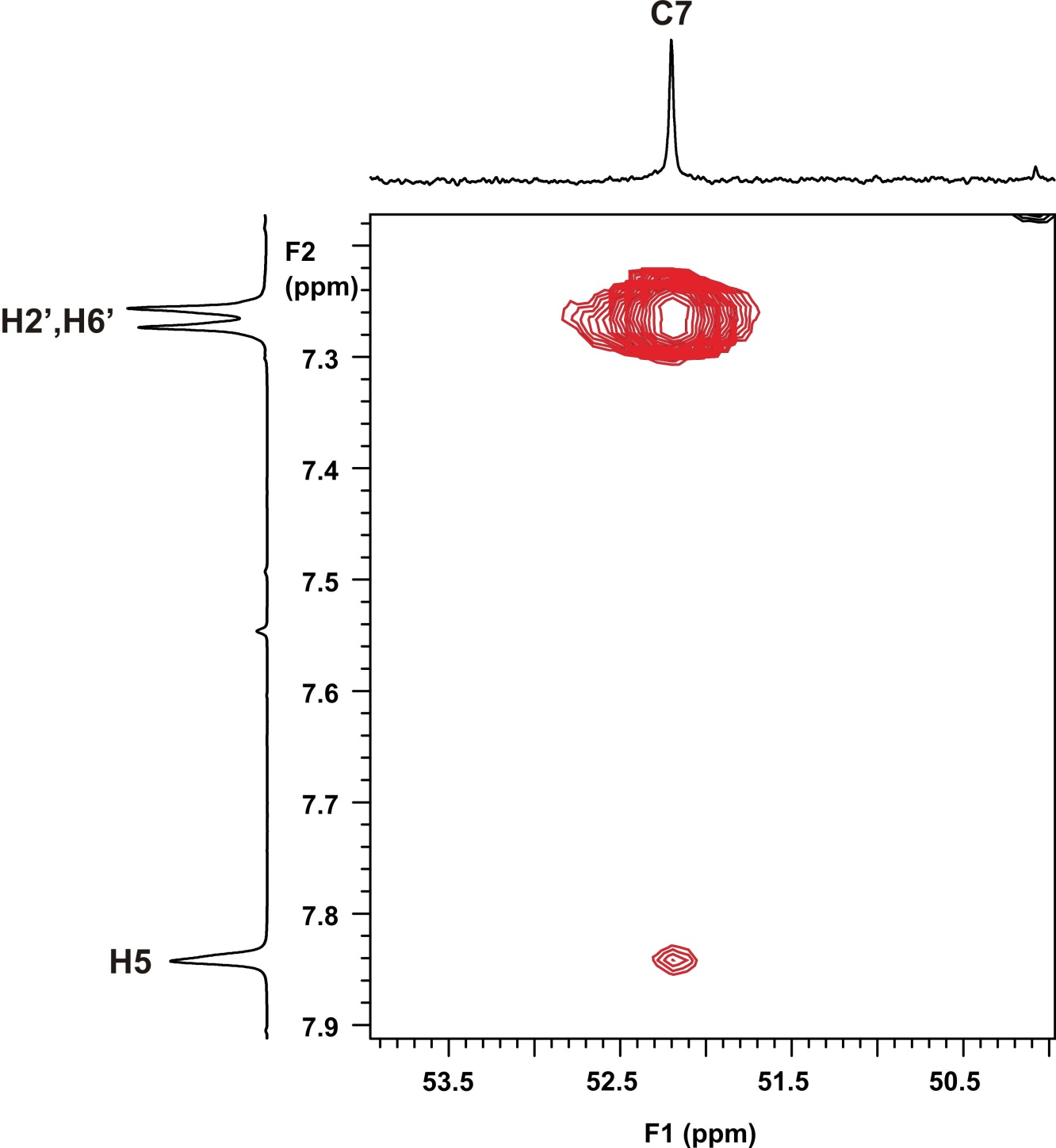


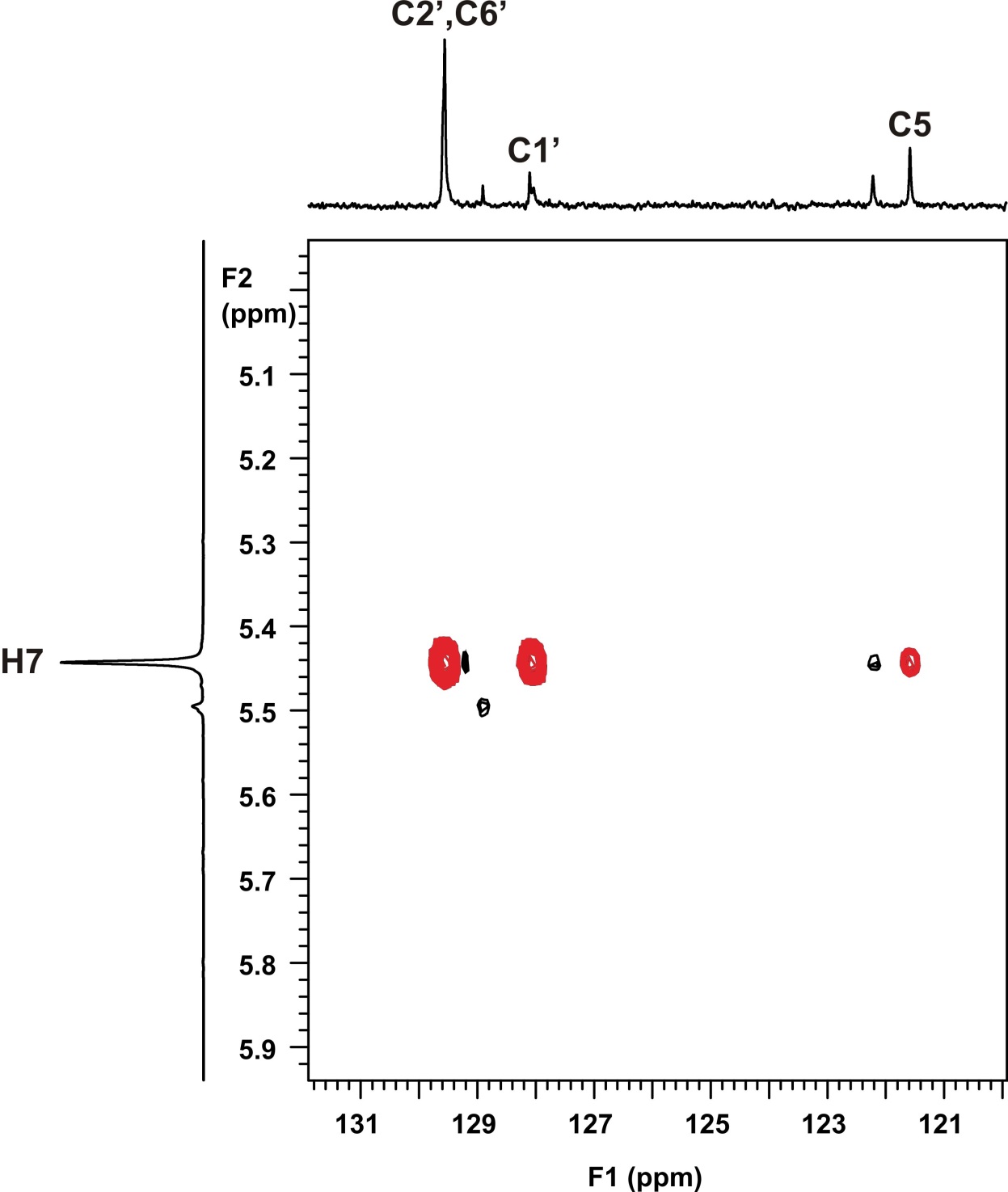


Fig. 6-SI. The part of ^1^H{^13^C} gHMBC spectrum of “reaction mixture of Step I-2” recorded at 298 K.


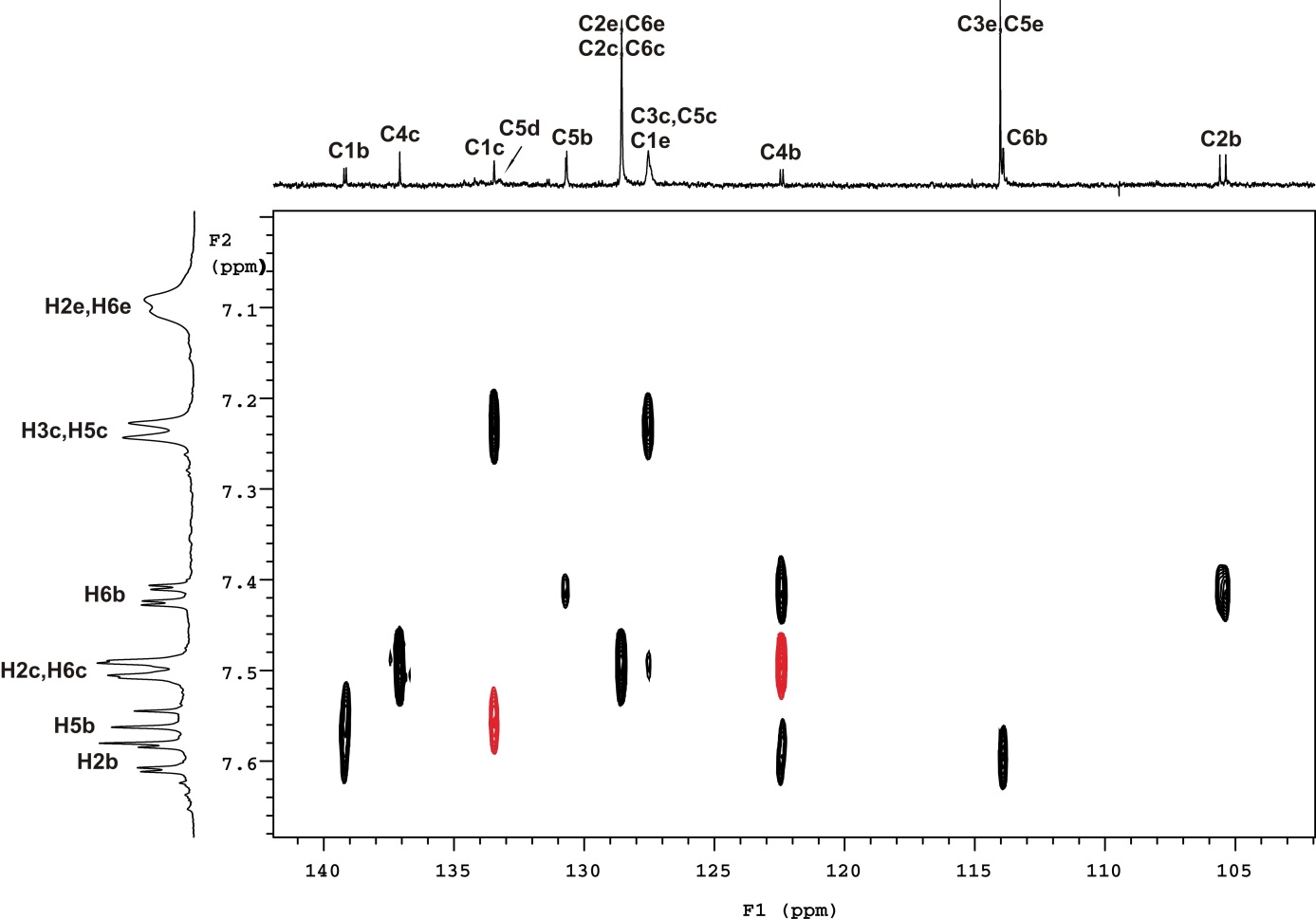


Fig. 7-SI. The part of the ^1^H{^13^C} gHMBC spectrum of compound **10** recorded at 298 K (cross peaks marked in red) demonstrated the direct bond between two aromatic rings of substrates **3** and **9** and consequently the formation of compound **10.**
